# Perceived Occupational Stress and Its Psychosocial Predictors Among Indian Orthodontists

**DOI:** 10.7759/cureus.52266

**Published:** 2024-01-14

**Authors:** Kiran K Kumari, Gowri Sankar Singaraju, Lakshmi Niharika, Greeshma M Harini, Ganugapanta Vivek Reddy, Prasad Mandava

**Affiliations:** 1 Orthodontics and Dentofacial Orthopaedics, CKS Teja Institute of Dental Sciences, Tirupati, IND; 2 Orthodontics and Dentofacial Orthopaedics, Narayana Dental College & Hospital, Nellore, IND

**Keywords:** mental health, practice, demographic, balance, stress, predictors, occupation, orthodontist, management

## Abstract

Background: Orthodontists, like any other medical professional, are susceptible to cumulative stressors and their undesirable consequences. The study aims to assess the self-perceived occupational stress levels and the psychological link between predictors and stress among orthodontic practitioners in India.

Materials and methods: The participants in this cross-sectional study are active members of the Indian Orthodontic Society (IOS). The data for the survey were collected by a previously validated closed-ended occupational stress assessment (OSA) questionnaire and a job satisfaction questionnaire, which were sent through the registered e-mails. A five-point Likert scale was used to assess the severity of individual stressors, and an overall severity score was obtained by summing up the individual scores. The predictors of stress based on socio-demographic parameters were assessed using a binomial multiple logistic equation. Statistical significance was set at a p-value of <0.05.

Results: A total of 311 responses were received. Male orthodontists, unmarried, in the age group of 30-40 years, working in urban areas without any academic attachment were more stressed compared to the other groups in the respective categories. Tiredness/headache (39%) was reported as the most common consequence of occupational stress. The most concerning stressor was patients not wearing retainers. Orthodontists showed overall job satisfaction that is negatively correlated to overall stress (p <0.0001)(r = -0.260).

Conclusion: A profound variation in stress levels was found among the orthodontists with their socio-demographic and professional characteristics. Despite the stress, orthodontists were highly satisfied with their careers.

## Introduction

Stress is a common phenomenon in dental practice. The highly technique-sensitive and rigorous nature of the work, dealing with non-compliant patients, a large workload, the repetitive nature of the work, anxieties, and fear about patients and their payments may all lead to stress in oral health professionals [1]. Apart from this, a dental practice requires precise treatment procedures that have to be completed within a definite time period, especially in the orthodontic specialty. This requires the orthodontist to be constantly focused on the schedule and resources, which inevitably leads to increased stress levels [2].

Occupational stress has been regarded as a personal, physical, and emotional imbalance in the workplace [3]. An unbalanced work-life schedule has been linked to several negative consequences that impact one's health and general well-being. Work-life conflict has been linked to low psychological well-being, depression, burnout, and high levels of stress [4]. The occupational stress of a dentist may have a negative impact on patients as well as ultimately lower the standard of care provided. Orthodontic practice comes with the challenges of working with patients for extended periods, managing specialists for treatment procedures, balancing work and personal life, and so on. Very few studies have been conducted to determine the stress levels of orthodontic practice.

Survey-based studies on occupational stress among orthodontists were conducted in Canada [3,5], the United Kingdom [6-8], Morocco [9], Italy [10], Australia/New Zealand [11], and Saudi Arabia [12]. A review of the literature reveals there were no published studies evaluating occupational stress among orthodontists in India. This study set out to explore occupational stress and job satisfaction among orthodontists in India. This survey was intended to identify the perceived sources and extent of occupational stress to evaluate the influence of different variables and job satisfaction on overall stress.

## Materials and methods

This was a one-phase cross-sectional study approved by the Institutional Review Board of Narayana Dental College & Hospital, Nellore, India (approval number: NDC/IECC/ORTHO/12-18/02). The sample for this study was drawn from the registry of the Indian Orthodontic Society (IOS), and the source is the IOS Member’s Directory 2017. The study included all life members who had at least three years of experience. All other categories of members such as affiliated, associated, and student life members, deceased members, and members practicing in other countries were excluded from the study. Around 3273 members who met the inclusion and exclusion criteria were included in the study. Assuming a confidence interval of 95% with a margin of error of 5%, the minimum sample size response required was estimated to be 344 to attain statistical significance.

Data collection

The data collection for the survey used a closed-ended questionnaire administered via Google Forms (Google LLC, Mountain View, California, United States) that was created and sent to the orthodontists via email. This survey was conducted between July 1, 2020, and September 30, 2020 with two reminders in between. The survey instrument formulated for the study was based on the Occupational Stress Questionnaire [13], validated on the orthodontist population [3,12], and the Dentist Satisfaction Survey [14]. The study used a slightly modified version based on the Indian context, adding three new categories to the original questionnaire. The questionnaire was divided into three parts after an excluder composed of two questions to avoid duplicates related to experience and registration number.

Socio-Demographic and Professional Characteristics

The first section of the questionnaire comprises 19 items related to respondents' demographic and professional information, such as gender, age, education, experience, academic attachment, type of clinical practice, and duration of hours spent on practice.

Occupational Stress Survey

The second part consisted of questions related to the occupational stress survey. The questions were originally listed based on stress-related categories such as patient, income, staff, time, and work-related. New domains including health, personal life, and professional work nature in India were additionally included to evaluate a total of 42 potential stressors. Participants were asked to mark the severity and frequency of the occurrence. A five-point Likert-type scale.was used ranging from "no stress" (a score of 1) to "very stressful" (a score of 5). The frequency was also scored on a five-point scale coded N, R, M, W, and D for Never, Rarely, Monthly, Weekly, and Daily, respectively. The subjects were provided with the option to select more than one answer for a particular question regarding the most significant consequence of occupational stress (Q10), and the most effective method to relieve occupational stress (Q11).

Overall Job Satisfaction Survey

Modified dentist satisfaction was used for evaluating overall job satisfaction [14]. A five-point Likert scale was used to assess overall job satisfaction using seven items, with scores ranging from "strongly disagree" (scoring 1) and strongly approve (scoring 5) serving as the assessment endpoints [15].

Statistical analysis

The data obtained from Google Forms was transferred and coded into a Microsoft Excel (2007) spreadsheet (Microsoft Corporation, Redmond, Washington, United States) for analysis. The data were then subjected to analysis by R Statistical Software v4.0.0 (2020; R Foundation for Statistical Computing, Vienna, Austria). The quantitative and categorical data were presented as mean and standard deviation, frequencies, and percentages. The presence or absence of stress in relation to job satisfaction and socio-demographic, working, and professional characteristics of orthodontists was assessed by multiple logistic equations. A p-value of less than 0.05 was considered to be statistically significant.

## Results

In total, 311 responses were received, and the overall response rate was approximately 10%. Of these, 27 responses were excluded due to incomplete survey filling, and a total of 284 responses were finally analyzed. The basic demographic details include the zone of practice, age, gender, years of practice, and type of practice of the IOS-registered orthodontists (Table 1). The majority of the study participants were males (62%), compared to females (38%). The majority of the orthodontists who responded belonged to the age group of 30-40 years (52%). Approximately 45% of the respondents had practiced for less than five years. The majority of orthodontists who responded were working in both educational institutions and private practice (49%), and only 10% were limited to academics. A major chunk of the orthodontists who responded had solo practice (n=183, 65%) and were from urban regions (n=174, 61%). Most of the orthodontists preferred working for four to eight hours.

**Table 1:** Contingency tables for the response variable stress (Y): presence or absence for different frequency categories of sociodemographic parameters Model fit: χ²(26) = 193.44; p = <0.001; Pseudo-R² = 0.66; Akaike information criterion (AIC) = 153; C-statistic = 0.979;  Hosmer-Lemeshow Test = Chi-sq.(8) 8.10 (p=0.424) *p< 0.05 is statistically significant

Descriptive Data			Analytical data					
Dependent: Stress (Y)	Group, n (%)	Stress scores, mean± SD	Within group, n (%)		OR (univariate) with CI	p-value	OR (multivariate) with CI	p-value
Independent variables			Score <2 (Stress absent)	Score >2 (Stress present)				
Zone - X1								
North - X1(1)	45 (15%)		7 (15.6)	38 (84.4)	-	-	-	
East - X1(2)	21 (7%)		7 (33.3)	14 (66.7)	-	-	-	-
West - X1(3)	58 (21%)		18 (31.0)	40 (69.0)	-	-	-	-
South - X1(4)	146 (52%)		28 (19.2)	119 (80.8)	-	-	-	-
Central - X1(5)	13 (5%)		-	13 (100.0)	-	-	-	-
Age (years) - X2								
< 30 - X2(1)	50 (18%)	2.6 (0.7)	5 (10)	45 (90)	Ref-cat		-	
30-40 - X2(2)	148 (52%)	2.7 (0.7)	10 (6.8)	138 (93.2)	1.52 (0.45-4.53)	0.464	2.79 (0.55-13.67)	0.205
40-50 -X2(3)	65 (23%)	2.4 (0.8)	26 (40)	39 (60)	0.17 (0.05-0.44)	0.001^*^	0.59 (0.13-2.60)	0.488
> 50 - X2(4)	21 (7%)	1.8 (0.3)	19 (90.5)	2 (9.5)	0.01 (0.00-0.05)	0.001*	0.01 (0.00-0.12)	0.001*
Gender - X3								
Male - X3(0)	177 (62%)	2.5 (0.7)	36 (20.5)	141 (79.5)	Reference category	-	-	-
Female - X3(1)	107 (38%)	2.7 (0.9)	24 (22.4)	83 (77.6)	0.89 (0.50-1.61)	0.693	4.40 (1.55-16.07)	0.016*
Marital Status - X4								
Unmarried -X4(0)	66 (23%)	2.9 (0.7)	3 (4.5)	63 (95.5)	Reference category	-	-	-
Married - X4(1)	218 (77%)	2.5 (0.8)	58 (26.3)	160 (73.7)	0.13 (0.03-0.38)	0.001*	0.47 (0.08-2.28)	0.377
Nature of workplace - X5								
Urban (metropolitan and corporate) - X5(1)	174 (61%)	2.7 (0.8)	15 (12.9)	101 (87.1)	Ref-cat	-	-	-
Non-Urban (municipality and less) - X5(2)	110 (39%)	2.6 (0.9)	20 (34.5)	38 (65.5)	0.28 (0.13-0.60)	0.001*	0.57 (0.16-1.98)	0.371
Years of practice - X6								
<5 - X6(1)	128 (45%)		-	-	-	-	-	-
6-15 - X6(2)	94 (33%)		-	-	-	-	-	-
16-25 - X6(3)	43 (15%)		-	-	-	-	-	-
>25 - X6(4)	19 (7%)		-	-	-	-	-	-
Academic attachment - X7								
Educational institute - X7(1)	28 (10%)	2.3 (0.6)	18 (15.3)	101(84.7)	Reference category		-	
Private practice - X7(2)	119 (41%)	2.6 (0.7)	6 (21.4)	22 (78.6)	1.52 (0.50-4.10)	0.430	2.63 (0.50-13.73)	0.249
Both - X7(3)	137 (49%)	2.5 (0.9)	36 (26.3)	101 (73.7)	0.77 (0.26-1.94)	0.592	1.42 (0.31-6.60)	0.650
Nature of practice - X8								
Restricted to orthodontics - X8(1)	131 (46%)	2.7 (0.7)	12 (9.2)	119 (90.8)	Reference category		-	
Both orthodontics and general - X8(2)	153 (54%)	2.4 (0.8)	49(31.6)	104 (68.4)	0.22 (0.11-0.42)	<0.001*	0.06 (0.01-0.25)	0.001*
Type of practice - X9								
Solo practice - X9(1)	183 (65%)	2.5 (0.8)	46 (25.0)	139 (75)	Reference category		-	
Group practice - X9(2)	83 (29%)	2.8 (0.8)	0	35 (100)	1.44 (0.67-3.38)	0.366	4.34 (1.10-20.24)	0.047*
Branded hospitals - X9(3)	18 (6%)	2.2 (0.3)	9 (18.8)	39 (81.2)	0.73 (0.25-2.43)	0.583	0.78 (0.18-3.85)	0.004
Days of work in a week - X10								
5 days a week - X10(1)	53(19%)	2.7 (0.5)	9 (17.0)	45 (83.0)	Ref-cat		-	
6 days a week - X10(2)	124 (44%)	2.4 (0.8)	40 (32.3)	84 (67.7)	0.24 (0.11-0.49)	<0.001*	0.45 (0.13-1.50,)	0.256
7 days a week - X10(3)	107 ( 37%)	2.8 (0.9)	11 (10.4)	95 (89.6)	0.57 (0.22-1.50)	0.241	3.16 (0.57-20.79)	0.203
Hours spent at work per day - X11								
<2 - X11(1)	57 (20%)	2.8 (0.7)	4 (7.0)	53 (93.0)	Reference category		-	
2-4 - X11(2)	69 (24%)	2.6 (0.6)	11 (15.9)	58 (84.1)	0.40 (0.11-1.24)	0.133	0.43 (0.08-2.18,)	0.341
4-8 - X11(3)	111 (39%)	2.5 (0.9)	34 (30.6)	77 (69.4)	0.17 (0.05-0.46)	0.002*	0.08 (0.01-0.35)	0.002*
>8 - X11(4)	47 (17%)	2.2 (0.5)	11 (23.9)	36 (76.1)	0.24 (0.06-0.76)	0.022*	0.96 (0.17-5.19)	0.965

To determine the mean score for each item, the respondents' scores for each of the individual 42 potential stressors were totaled. All the potential stressors with a mean severity score greater than 3 were ranked in order (Table 2). The stressor ‘patients not wearing retainers’ had the highest mean severity score of (3.6±1.1). The mean individual scores for the eight categories of stressors are presented in Table 3. The mean score with the maximum value was ‘patient-related stressors’ with a mean score of 3.14+ 1.19. ‘Time-related stressors’ has the smallest mean score of 1.59+0.66. The association and correlation between the individual socio-demographic factors and working conditions and the presence or absence of stress were assessed by the odds ratio by regression through a general block model (Table 1).

**Table 2:** Most concerning stressors in orthodontics: stressors with mean severity and mean frequency scores

S. no	Potential stressor	Mean severity score		Mean frequency	
		Mean	SD	Mean	SD
1.	Not wearing retainers	3.6	1.1	3	0.9
2.	Patients with broken appliances	3.5	1.2	2.1	0.9
3.	Uncooperative patient	3.4	1.1	2.8	0.9
4.	Relapse in retention patients	3.3	1.2	2.2	0.7
5.	Dealing with unrealistic patient expectations	3.2	1.2	2.6	0.9
6.	Motivating patients with poor oral hygiene	3.2	1.2	2.7	0.9
7.	Inability to meet my expectations	3.2	1.4	2.3	1.1
8.	Treating a case with an unfavorable prognosis	3.2	1.2	2.2	0.7
9.	Repayment of loans	3.2	1.4	2.3	1.1
1.	Demanding pre-removal of the appliance	3.1	1.2	2.6	0.9
11.	Accepting compromised treatment results	3.1	1.2	2.4	0.7
12.	Assuming a heavy financial burden	3.1	1.4	2.3	1.1
13.	Quoting and collecting fees	3.1	1.2	2.6	1.1
14.	Making enough money to cover overhead expenses	3.1	1.4	2.5	1.1
15.	The patient shows dissatisfaction with the care received	3	1.3	2.2	0.9
16.	Long work hours	3	1.3	2.3	1
17.	Managing paperwork	3	1.2	2.6	1

**Table 3:** Mean category scores for the eight categories of stressors

S. No	Stressor category	Number of items in the category	Mean severity score	SD	Max	Min	Median
1	Patient-related	10	3.14	1.19	5	1	3
2.	Income-related	4	3.10	1.33	5	0.5	3
3.	Work-related	9	2.77	1.23	4.88	1	2.55
4.	Staff related	5	2.72	1.08	5	1	2.8
5.	Professional related	3	2.54	1.20	5	0.66	2.33
6.	Personal	3	2.3	1.15	5	1	2.33
7.	Health-related (After the start of practice)	4	2.1	1.35	5	0.75	1.5
8.	Time-related	3	1.59	0.66	3	0.6	1.6
	Overall stress		2.54	1.15	4.73	0.81	2.39

The consequences of the stress are shown in Table 4. The orthodontists reported 'tiredness/headache' (n=170, 39%) as a consequence of stress followed by 'anger/frustration' (n=118, 27%). Most orthodontists reported spending time with family and friends (n=229, 48%) as a reliever of stress, followed by physical exercise (n=101, 22%) (Table 4). Binomial logistic regression was carried out to analyze the combined effect and interaction of the socio-demographic variables on stress generation (Table 5). A 10-factor predictor logistic model was generated after the model was run in 'Entry' mode, allowing block regression of all the variables.

**Table 4:** Perceived consequences of stress and reported methods of stress relaxation *The total combination may exceed than total respondents (n=284) if more than one option was selected by the individual respondent.

Consequence	Frequency (n*)	Percentage
Nervousness	40	9%
Tiredness/headache	170	39%
Anger/frustration	118	27%
Fatigue and exhaustion	89	20%
Depression	22	5%
Methods of stress relief		
Meditation	58	12%
Social media	58	12%
Spending time with family and friends	229	48%
Physical exercise	101	22%
Attending Stress-managing programs	9	2%
Others	20	4%

**Table 5:** Multiple logistic regression model for prediction of stress with job satisfaction and sociodemographic and professional characteristics Method: Enter Block regression; Model fir: χ²(21) = 148.155, p = <0.001; Model summary -2 Log likelihood-144.248;  Cox & Snell R Square-0.408; Nagelkerke R Square-0.633; Hosmer-Lemeshow test = Chi-sq.(8) 4.916 (p=0.767) *p< 0.05 is statistically significant

Dependent variable: absent-0; stress present-1		Regression parameter			Sig.
Independent variable (X)		B	Wald	Log(B)	
Step 1	Overall job satisfaction	-0.959	3.318	.383	0.06
	Age (years) - X2		20.989		0.00*
	< 30 - X2(1)	4.640	9.989	103.534	0.02*
	30-40 - X2(2)	5.567	16.968	261.723	0.00*
	40-50 - X2(3)	3.705	7.923	40.655	0.05*
	Female gender - X3(1)	-1.274	4.081	.280	0.04*
	Marital status - X4		.517		0.77
	Unmarried - X4(0)	-12.840	.000	.000	1.00
	Married - X4(1)	-12.207	.000	.000	1.00
	Nature of workplace - X5		.219		0.89
	Urban (metropolitan and Corporate) - X5(1)	20.187	.000	584862707.776	1.000
	Non-Urban (municipality and less)	0.256	.219	1.292	0.64
	Academic attachment - X7		4.946		0.08
	Educational institute - X7(1)	1.142	3.459	3.132	0.06
	Private practice - X7(2)	-.562	.422	.570	0.51
	Nature of practice - X8		12.901		0.02*
	Restricted to orthodontics - X8(1)	-15.066	.000	.000	1.00
	Both orthodontics and general -X8(2)	-17.956	.000	.000	1.00
	Type of practice X9		5.353		0.06
	Solo practice - X9(1)	1.010	1.378	2.746	0.24
	Group practice - X9(2)	2.545	4.972	12.742	0.02*
	Working days in a week - X10		9.025		0.03*
	5 days a week - X10(1)	2.598	4.206	13.442	0.04*
	6 days a week - X10(2)	1.053	1.239	2.867	0.26
	7 days a week - X10(3)	2.862	6.096	17.501	0.01*
	Hours spent at work per working day - X11		15.894		0.00*
	<2 - X11(1)	-.311	.101	.733	0.751
	2-4 - X11(2)	-1.216	2.517	.296	0.13
	4-8 - X11(3)	-2.842	12.102	.058	0.01*
	Constant	29.447	.000	6146.98	1.00

## Discussion

Orthodontists, like any other medical profession, are susceptible to cumulative stressors and their undesirable consequences. It is crucial to identify the factors that influence stress management behaviors to help them cope with the stress that is encountered daily. Even though occupational stress is not as high as it is in surgical specialties, occupational stress in orthodontic clinical settings has been linked to better treatment outcomes and job satisfaction. Professional implications of stress include absenteeism, decreased productivity, and work discontent [14]. The working burnout in orthodontic practice can be reduced if these perceived sources of occupational stress are identified at an early stage. Occupational stress in orthodontics is not well researched, and to the best of our knowledge, there are no such studies among orthodontists in India. Therefore, this study was undertaken to determine the self-perceived potential stressors and their magnitude in relation to socio-demographic characteristics among Indian orthodontists. A total of 311 responses were received, of which 284 were analyzed.

Potential stressors: mean severity score

A total of 42 potential stressors were classified into eight domains: income, patient, time, staff, work-related, health, personal life, and professional work (Table 2). A mean score of severity ≥ 3.0 was noted for the analysis of the 17 items of the total 42 potential stressors. About 79% of orthodontists in our study perceived stress with mean scores above 2. The overall stress had a mean stress score of 2.54 ±1.15, a maximum stress score of 4.73, a minimum stress score of 0.81, and a median of 2.39. The mean severity scores of the following stressors have shown the highest stress scores: ‘patient not wearing retainers‘ (3.6±1.1), ‘patients with broken appliances‘ (3.5±1.2), and ‘uncooperative patient’ (3.4±1.1). It was noted that factors such as cardiovascular diseases, nervous disorders, and family life were found to be less stressful, with mean scores less than 2. The mean stress values in the present study are of lesser magnitude compared to those of Canadian orthodontists [3]. In the Canadian study, the three stressors that earned the highest scores were patient-related, such as patient dissatisfaction with therapy (3.82), working with a challenging or uncooperative patient (3.75), and failure of patients to keep an appointment schedule (3.56). A stressor among orthodontists in Morocco was the patient’s discontent towards the treatment given, with a mean of 4.04± 1.09, and interpersonal communication with the staff was rated as having the lowest severity (3.02±1.14) [9].

The elements influencing the work-life balance of orthodontists in the United Kingdom were standing up to the expectations of the patient, having a rigid work schedule, being able to work part-time, and being able to take sudden leaves of absence [6]. On the Asian side of the globe, a study in Saudi Arabia revealed that the main issues of orthodontists are too much paperwork and patients’ poor adherence to their appointment times [12]. In the present study, the most concerning stressors with the combined highest mean severity and mean frequency scores were patients not wearing retainers (3.6, 3.0), uncooperative patients (3.4, 2.8), motivating patients with poor oral hygiene (3.2, 2.7), dealing with unrealistic patient expectations (3.2, 2.6), and demand by patients for pre-removal of the appliance (3.0, 2.0) (Table 2). The findings indicate that most orthodontists see these events as stressful, and they may happen more frequently than once per month.

Potential stressors: mean domain score

‘Patient-related’ stressors were accorded the highest scores (3.14±1.19) among the eight categories of stressors (Table 3). The lowest scores were related to time-related stressors, which may be because time-related factors were given less importance in India. This was in contrast to the study of Alqahtani on Saudi Arabian orthodontists [12]. In their study, patient-related stressors were ranked the highest (3.38) followed by work and time-related stressors with mean scores of 3.36 and 3.32, respectively. Referral and income-related stressors (2.39) were the least concerning ones. In Morocco, high average frequency and severity are factors that are related to the patient (3.74 ± 0.78) and those related to time (3.07 ± 0.87) [9]. The findings imply that to reduce their level of occupational stress, clinicians should first think about strategies to strengthen their ability to manage both their time and patients effectively. In the current study, the orthodontists reported tiredness/headache (n=170, 39%) more as a consequence of stress followed by anger/frustration (n=118, 27%), and fatigue and exhaustion (n=89, 20%). The most popular methods for stress management found in the current study were spending time with family and friends (n=229, 48%), while the least commonly used were attending stress-managing programs (n=9, 2%). Most orthodontists reported the other relievers of stress as physical exercise (n=101, 22%), meditation (n=58, 12%), and social media (n=58, 12%).

Overall job satisfaction

The mean score of overall job satisfaction in this study is 3.59±0.62, with a maximum value of 4.57, a minimum value of 1.85, and a median of 3.71. The attitude of the person towards his profession is reflected in job satisfaction levels. Most orthodontists said that if they had to choose again, they would pursue a profession in orthodontics (n=234, 82%) and were satisfied with the profession. This was in agreement with the findings of earlier studies on Canadian orthodontists (80%) [3] and a study on Britain orthodontists (83%) [7], who expressed satisfaction with their choice of profession. Univariate regression analysis revealed that stress and overall job satisfaction are negatively correlated. Similarly, an overall satisfaction of 80.7% was conveyed by orthodontic professionals in Saudi Arabia, and only 4.4% reported dissatisfaction [15]. 

Association between stress and socio-demographic parameters

The individual scores of stress carry less meaning than being categorized as having stress or not, so the dependent variable was converted into dichotomous scores. The stress score above the value of 2 is marked as 'stress present,’ and that below 2 is marked as ‘no stress.’ The multicollinearity between the variables was checked by the variance inflation factor (VIF). A significant multicollinearity was found between years of experience and age. The age was kept, and the years of experience were eliminated in the final model. There was similar multicollinearity in some of the categories of type of practice: solo/group. In the final model, the group practice and partnership practice were combined.

Logistic regression analysis

A legitimate research hypothesis posed to the data was that the likelihood that an orthodontist may develop stress is related to his/her age, gender, marital status, nature of the practice, number of days work in a week and number of hours they spent on orthodontic work and the overall job satisfaction. We made a model with stress as a categorical dichotomous variable and nine predictors that were based on the participants' socio-demographics, working and occupational characteristics, and professional profiles (Table 5).

According to this model, the log of the odds of orthodontist having stress is positively related to Age (X2): X2(1)<30 years, Age X2(2): 30-40 years, and Age X2(3): 40-50 years; Nature of workplace (X5)-X5(1): Urban setting; Acad attachment (X7): X7(1) only in academics; Type of practice X9: X9(2) group practice; Working days in a week (X10): X10(1) five days in a week, X10(2) six days in a week; X10(3) seven day in a week (p < 0.05).

The log of the odds of orthodontist having stress is negatively correlated to overall job satisfaction; Gender (X3): X3(1) female; Marital status (X4): X4(0) Unmarried, X4(1) Married; Academic attachment (X7): X7(2) only in Private practice; Nature of practice (X8): X8(1) Orthodontist only, X8(2) Orthodontist and general; Hours of work per working day (X11): X11(1) <2 hours, X11(2) Two to four hours per day, X11(3) four to eight hours per day. The percentage by which the estimated OR decreased is statistically significant (p < 0.05) (Table 5).

The soundness of a logistic regression model was also assessed, and the logistic model at step 1 was more effective than the null model. The results of the Omnibus Tests of Model Coefficients reveal that the new model is much better, indicating that the addition of the explanatory variables increased the model's accuracy (chi-square=148.155, df =21, p<0.000). Nagelkerke’s R² is 0.633, which indicates that the model is good and also efficient. The test for goodness of fit shows a p-value of 0.767, and it is not statistically significant, indicating the fitness of the data of the variables fitted into the model. The prediction of the new model is 94.7%.

This survey is the first of its kind that identified the most concerning stress-related issues in orthodontics based on the high frequency and severity of their occurrence in their job. The information acquired in this study can be used by orthodontists in India as a major guideline for patient care in orthodontic practice. This survey provides data for further studies for evidence-based information about occupational stress among Indian orthodontists. as well as to compare with other specialties and dentistry in general.

When evaluating the findings of this study, a few limitations should be considered. The main drawback of our survey is the low response rate, which falls short of 5% compared to the estimated response rate. Initially, the survey was designed as a direct face-to-face interview or self-administered paper format method. The direct interview type of study was conducted initially at the national annual conferences of the IOC held at Cochin, in 2018, and at Bhubaneswar, in 2019. We got only 20 direct responses and 35 paper-based surveys were returned out of a total of 2200 orthodontists registered for these conferences. The participants in the direct interview were reluctant to answer some of the questions that were related to their mental health status. This made the individual data recorded invalid. It is for this reason, considering the size of the population and to increase the response rate of the study population, an e-mail survey was conducted for three months.

Another shortcoming of the study is the lengthy nature of the questionnaire. This may also be one of the reasons for the low response rate. But, considering the comprehensive nature of the problem under study, this was unavoidable. However, this was overcome by conducting a pilot study and our survey instrument was verified against the objectives by a direct interview of 10 locally practicing orthodontists with more than three years of experience in clinical practice and willing to volunteer and enable us to check the flow and skip patterns. The time for answering all the questions was found to be 10-15 minutes with an average time of 7.5 minutes. The contents of the questionnaire were peer-reviewed by an external orthodontist professor with 20 years of experience in private practice not related to the study and a senior biostatistician associated with healthcare studies. Necessary modifications were made so that the final tool formulated collected the data with the required accuracy and reliability. Another reason for non-response may be loss of interest. Respondent’s boredom and fatigue were counteracted by fractioning the questionnaire into sections and each section was administered separately.

Considering the above shortcomings and the important insights obtained from the study, the national professional bodies or the research organizations involved in the population studies should come forward to conduct such studies organizationally. There are more than 5000 registered life members of the IOS and every year around 1000 new members are being added [16]. Indian orthodontists comprise one of the largest workforce next to the United States [17,18]. This requires expanding, diversifying, and stratifying the sample size as there is a wide geographical distribution of the participants in our study.

The study's cross-sectional design and the results of the study only represent data from questionnaires, which is a notable limitation. Further research into the long-term effects of occupational stress is needed. As a result, longitudinal studies are critical for investigating changes in workplace environmental elements and should be conducted future.

## Conclusions

The assessment of occupational stress and identification of potential stressors among Indian orthodontists revealed considerable heterogeneity. The findings of this study may have an impact on how orthodontic therapists build their career paths and provide guidance for workforce planning procedures. Those in orthodontic practice can prevent occupational stress for themselves and their team by exercising caution, recognizing it, and planning ahead of time. It is necessary to lead a healthy lifestyle and take preventative steps to lessen stress. Clinical competence and effective orthodontic practice management continue to be a true cornerstone for decreasing stress in everyday orthodontic practice
